# Genital *Chlamydia trachomatis* Seroprevalence and Uterine Fibroid Development: Cohort Study of Young African-American Women

**DOI:** 10.3390/microorganisms10010010

**Published:** 2021-12-22

**Authors:** Kristen Moore, Donna Baird

**Affiliations:** Epidemiology Branch A3-05, National Institute of Environmental Health Sciences, Research Triangle Park, Durham, NC 27709, USA; kristen.moore@nih.gov or

**Keywords:** *Chlamydia trachomatis*, seroprevalence, uterine fibroids, incidence, tumor growth

## Abstract

Few studies have investigated the 1930s hypothesis that reproductive tract infections are risk factors for fibroid development. In our 2017 cross-sectional analysis from the Study of Environment, Lifestyle, and Fibroids (2010–2018), a large Detroit community-based cohort of 23–35 year-old African-American women with ultrasound fibroid screening, we found an inverse association between seropositivity for genital *Chlamydia trachomatis* (gCT) infection and fibroids. With prospective data from the cohort (standardized ultrasounds every 20 months over 5 years), we examined gCT’s associations with fibroid incidence (among 1158 women fibroid-free at baseline) and growth. We computed adjusted hazard ratios (aHRs) and 95% confidence intervals (CIs) for incidence by gCT serostatus using Cox proportional hazards models. GCT’s influence on growth was assessed by estimating the difference between fibroid size change for seropositive vs. seronegative between successive ultrasounds (1254 growth measures) using a linear mixed model. Growth was scaled to change over 18 months. GCT seropositivity was not associated with fibroid incidence (aHR, 1.0 95% CI: 0.79, 1.29) or growth (4.4%, 95% CI: −5.02, 14.64). The current evidence based on both biomarker gCT data, which can capture the common undiagnosed infections, and prospective ultrasound data for fibroids suggests that *Chlamydia* is unlikely to increase fibroid risk.

## 1. Introduction

Uterine fibroids, common benign smooth muscle cell tumors, are one of the most common gynecologic conditions affecting women during their reproductive years, with estimated total annual costs in the United States (US) of up to $34 billion [1]. Symptoms resulting from fibroids (pain, severe menstrual bleeding, reproductive problems) are the leading indication for hysterectomy in the US. Black women are 2–3 times more likely to have fibroids than other race/ethnicity groups and have an approximately 10-year earlier age of onset than White women [2,3,4]. They also have larger and more numerous tumors at diagnosis [5] and thus are at higher risk of surgical/radiological treatment [6].

Fibroids are hormone dependent; they develop in premenopausal women and tend to regress after menopause [7]. Despite the high morbidity and public health costs, fibroids are an understudied condition with few well-established risk factors [8]. In addition to Black heritage, other established risk factors are older age (up to the age of menopause), younger age at menarche, nulliparity, and longer time since last birth [3,9,10]. Three studies have reported that the progestin-only injectable (i.e., Depo-Provera) is inversely associated with fibroids [9,11,12]. Other possible risk factors studied show inconsistent associations and/or have undergone limited study.

Witherspoon and Butler hypothesized in the 1930s that reproductive tract infections play a role in uterine fibroid development [13]. Reproductive tract infections could plausibly increase the risk of fibroids by inducing an inflammatory environment leading to cell proliferation, increased extracellular matrix production, and decreased apoptosis, all features of fibroid development [14,15]. However, of the few studies on this topic, most have used self-reported reproductive tract infection status, which can be plagued by recall error and misclassification due to the frequent asymptomatic nature of most reproductive tract infections.

In 2017, we published data using serology to identify prior genital *Chlamydia trachomatis* (gCT) infections that did not support the Witherspoon and Butler hypothesis [16]. Serology, the identification of antibodies in the serum that remain after infection, provides a highly sensitive and specific measure for past, cumulative exposure and, although literature is limited [17], there is a consensus from experts in the field that *C. trachomatis* antibodies persist years after infection and potentially for a lifetime. Contrary to the hypothesis, we found an unexpected inverse association between gCT serology and prevalent fibroids identified by ultrasound screening [16]. Inverse associations were similar across categories of fibroid size, number, and total volume. We focused on gCT because: it is one of the most common reproductive tract infections in the US, it has been isolated from the upper genital tract [18,19,20], causes pelvic inflammatory disease, and the prior studies based on self-reported diagnoses had inconsistent findings [3,21,22].

However, given the cross-sectional design of the prior analysis [16], the timing of fibroid onset in relation to gCT acquisition was unknown. We now have prospective data from the same cohort to investigate the association between gCT serology and subsequent ultrasound-documented fibroid development. We analyzed fibroid incidence from 5 years of ultrasound follow-up among women who were fibroid-free at their baseline examination, and fibroid growth among women who had fibroids present at baseline or developed them during the study.

## 2. Materials and Methods

The Study of Environment, Lifestyle, and Fibroids (SELF) was designed to prospectively study fibroid incidence and growth among a community-based volunteer sample of 23- to 35-year-old, self-identified African-American or Black women (*n* = 1693) in the Detroit, Michigan, area recruited between 2010–2012 [23]. Women were ineligible for the study if they: had a prior clinical diagnosis of uterine fibroids, a hysterectomy, a history of cancer treated with radiation or chemotherapy, or were on medication to treat lupus, Grave’s disease, Sjogren’s scleroderma, or multiple sclerosis. At four clinic visits approximately every 20 months over 5 years, participants provided questionnaire data, non-fasting blood samples; standardized study ultrasound examinations were conducted at each visit [23]. The study was approved by the institutional review boards of the National Institute of Environmental Health Sciences and Henry Ford Health System and all participants gave written informed consent.

### 2.1. Chlamydia Measurement

The presence of immunoglobulin G antibodies to gCT was assessed on 1661 baseline serum samples (98%) by the University of Washington Chlamydia Laboratory (Seattle, WA, USA) using the gold standard, species-specific micro-immunofluorescence assay [24]. Assay details were provided previously [16]. In brief, the laboratory’s antigen panel included purified elementary bodies of *Chlamydia trachomatis* (serovars A, B, I, and H and groupings of serovars CJ, DE, KL3, FG, and L1L2), *Chlamydia pneumoniae* (serovar TW183), and *Chlamydia psittaci* (avian strain 6BC). Participants who tested positive (1:16 dilution) for any of the gCT serovars B, I, H, CJ, DE, KL3, and FG were considered seropositive for gCT infection. A negative antigen control was included with each set of antigens on the micro-immunofluorescence slides, and a positive serum control analysis was run with each set of sera on a given day. *C. psittaci* was included as a genus control in the antigen panel to monitor species cross-reactive antibody responses. Those testing positive for *C. psittaci* may not have had a prior *C. psittaci* infection. Seventy-four samples with identical titers for gCT and *C. psittaci* were excluded. 

### 2.2. Fibroid Measurement

Certified sonographers assessed fibroids at visits by transvaginal ultrasound with a standardized protocol [23]. The 3 diameters (longitudinal (L), anterior-posterior (A), and transverse (T)) of fibroids ≥0.5 cm in at least one diameter were measured in 3 separate passes through the uterus. Fibroid volume (cm^3^) was calculated by computing the three triplicate volumes with the ellipsoid formula (L × A × T × 0.5233) and averaging across them [23]. The few instances of sonographer-noted visualization problems (<0.5%) were excluded. Details of ultrasound methods have been described elsewhere [23].

### 2.3. Covariates

Models were adjusted for the following covariates a priori based on the available literature: age, use of the injectable contraceptive, depot medroxyprogesterone acetate (DMPA) within the last 2 years (yes/no), recent birth (<5 years ago) (yes/no), number of births prior to last (0–1, 2, 3+) (all also had strong protective associations found with fibroids in this study population), current smoker (yes, no), age at menarche (modelled on an ordinal scale: ≤10, 11, 12, 13, 14+), and household income ($0–$20,000, >$20,000–$50,000, >$50,000) as a measure of socioeconomic status. Two other covariates were examined for potential confounding effects: current use of oral contraception (yes, no) and body mass index (BMI) kg/m^2^ (18.5 to <25, 25 to <30, 30 to <35, 35 to <40, 40+). Covariates were updated at each study visit except for age at menarche which was captured at baseline. The information on births was anchored to the end of each study interval as an efficient way to capture the strong inverse association with fibroids for births during an interval between visits; all other covariates were anchored at the beginning of the interval [25]. Additional factors to describe the cohort are listed in Table 1.

### 2.4. Statistical Analysis

#### 2.4.1. Fibroid Incidence

Fibroid incidence was defined as ultrasound detection of a fibroid at a follow-up visit for a woman who was fibroid-free at the enrollment ultrasound. Out of the 1587 participants with unequivocal gCT serology, 1158 (73%) were fibroid-free at baseline with follow-up ultrasound data. To examine the association of gCT with fibroid incidence, we estimated hazard ratios (HRs) and 95% confidence intervals (CIs) with Cox models (age was the timescale). We adjusted for the a priori factors stated above (years since last DMPA use, recent birth, number of births prior to last, income, current smoking, and age at menarche). BMI and birth control use were also evaluated but did not change the association, so they were not included in the final model.

#### 2.4.2. Fibroid Growth

Fibroid growth was estimated for individual fibroids identified as the same fibroid across two successive ultrasound visits based on the position in the uterus and/or by assessments of the archived ultrasound videos and images by the head sonographer. There were 1254 growth measurements from 395 women with gCT results. Growth was estimated by finding the difference in the natural log volume of matched fibroids across successive visits and scaling that to 18 months (dividing by the days between visits to get daily growth and multiplying by 540 for 18-month growth). This timeframe of a year and a half was chosen because it is close to our median interval between visits (19 months with IQR 18–21) and would be clinically meaningful.

We examined the influence of gCT serostatus on fibroid growth over the three follow-up intervals using a linear mixed model (GLIMMIX procedure in SAS 9.4) with a random intercept for participant and fibroid. This accounted for possible correlations between fibroids from the same woman and fibroids that were followed across more than one study interval (the time between one visit and the next). For ease of interpretation, we rescaled the log volume change for gCT-exposed fibroids vs. gCT-unexposed fibroids to an estimated percent difference in growth per 18 months by using the resulting beta from the regression and applying the formula [exp(β) − 1] × 100. As an example, an estimated percent difference of 10% indicates that the average volume change per 18 months for the exposed was an estimated 10% greater than the volume change of the unexposed fibroids. For this analysis, in addition to the a priori factors stated above (age, years since last DMPA use, recent birth, number of births prior to last, income, current smoking, and age at menarche) we also adjusted a priori for fibroid-related factors: fibroid volume (cubic centimeters, cm^3^) (<0.52, 0.52 to <4.19, 4.19 to <14.1, 14.1+) and fibroid number on an ordinal scale (1, 2, 3, 4+) at the start of the interval [26]. BMI and oral contraceptive use were also evaluated but did not change the association, so they were not included in the final model.

### 2.5. Sensitivity Analyses

For both fibroid incidence and growth, we performed two sensitivity analyses to restrict the data to subsets in which few women would seroconvert during follow-up after baseline gCT serology was captured, thus limiting any exposure misclassification. We restricted to 1) the first follow-up visit and 2) women aged 30+ years at baseline because serostatus changes little after age 30 [27]. Additionally, for growth, to evaluate the impact of outliers, we excluded statistical outliers defined as Studentized residuals > +/−3.

Analyses were conducted with SAS 9.4 (SAS Institute, Inc., Cary, NC, USA).

## 3. Results

### 3.1. Fibroid Incidence

Of the 1158 fibroid-free women at baseline, *n* = 277 (24%) had an incident fibroid and *n* = 682 (59%) were gCT seropositive. Compared to women seronegative for gCT, those seropositive tended to have a lower income, have a lower education level, have higher BMI, be current smokers, have higher numbers of sex partners, have a lower age at first sex, and be HSV-2 seropositive (Table 1). 

A similar proportion of those seropositive (23%) developed fibroids as those seronegative (25%). In the adjusted multivariable model, gCT was not associated with an increased risk of fibroid incidence (aHR: 1.0 95% CI (0.79, 1.29)) (Table 2). Sensitivity analyses did not impact the primary results (restricted to first follow-up visit, aHR: 1.2 95% CI (0.77, 1.72) and women aged 30+ years, aHR: 1.1 95% CI (0.76, 1.48)).

### 3.2. Fibroid Growth

The average growth per 18 months for the sample of intervals with available growth measurements (*n* = 1254) was an increase in volume of 77.5% (95% CI: 68.69, 86.86). Of the available growth measurements, *n* = 609 (49%) were among gCT seropositive women. The average growth for fibroids in seropositive women was an estimated 4.4% (95% CI: −5.02, 14.64) greater than in fibroids from the seronegative women (Table 3).

For the sensitivity analyses, when restricting to the first follow-up visit and women aged 30+ years there was no impact on the primary results (0.3% (−16.48, 20.38) and 1.6% (−9.63, 14.20), respectively). When removing 15 outliers, the growth estimate was 8.0% (95% CI: −1.15, 18.01).

## 4. Discussion

In this first prospective study on the relationship between gCT, as assessed by gold-standard serology, and fibroids, we did not find an increase in incidence or growth of fibroids among gCT seropositive women compared to gCT negative women. These findings differ from the suggestive protective associations for fibroid presence, size, number, and total volume seen in the previous cross-sectional analysis of prevalent fibroids at baseline in this same population [16]. The essentially null findings in the current analysis are based on a prospective study design, less subject to bias than the earlier cross-sectional analysis. The three other previous studies [3,21,22] had varied findings, were cross-sectional in design and relied on self-reported diagnosis of gCT which is problematic due to the high prevalence of undiagnosed infection [28]. The Faerstein study found suggestions of positive associations for those self-reporting a *Chlamydia* diagnosis (although with wide confidence intervals) [21], the Laughlin study showed null results for Black women and positive for White women (although with wide confidence intervals) [3], and our self-reported study showed inverse associations [22]. In our cross-sectional serology study, when categorizing seropositivity into 2 categories, those with a self-reported *Chlamydia* diagnosis (most likely symptomatic and potentially those with more of an inflammatory response) and those without a diagnosis, we found very little difference in estimates [16].

A limitation of our study is that we only had serology data at baseline, thus we could not look at seropositivity trends over time. We did collect self-report data at each visit to examine subsequent diagnosed infections, but too few women reported new infections during the prospective study to analyze those data [22]. Additionally, our sample is a volunteer sample of women; however, they were recruited by numerous community methods, and seroprevalence of gCT in our cohort (59%) was very similar to NHANES among 18- to 39-year-old African-American women (60% seropositive on both assays conducted) [29].

Finally, our study could have missed gCT influence on fibroid development if its effects operated in a “hit-and-run” manner. For example, if a gCT infection had immunologic effects that rendered normal myometrial tissue or existing fibroids either more or less susceptible to fibroid initiation and growth only during the infection, this would be difficult to evaluate even with our current detailed prospective study design. To assess such activity in an epidemiologic study would require frequent periodic testing of participants for active gCT infections throughout a multi-year longitudinal study of fibroid development. This was beyond the scope of our current study but might be possible in the future with monthly self-collection of vaginal swabs [30] that could be mailed into study staff, stored frozen, and analyzed in batches for acute active gCT infections [31,32].

Although further study is warranted, neither our prior cross-sectional findings suggesting a protective association [16], nor the current, detailed prospective analyses based on gCT serology showing null associations, support the general hypothesis that reproductive tract infections are risk factors for fibroid development. The evidence to date suggests that *Chlamydia* infections are unlikely to be an important risk factor for fibroid incidence or growth and are unlikely to explain the disproportionately higher fibroid burden in U.S. Black women. 

## Figures and Tables

**Table 1:** Selected characteristics of fibroid free black women aged 23–35 years according to *Chlamydia* serostatus at baseline (*n* = 1158).

	Chlamydia Serostatus		Total
Baseline Variable	No *n* = 476 *n* (%)	Yes *n* = 682 *n* (%)	1158
Age (years): median (IQR)	29 (26–32)	29 (26–32)	
Income			
<20 K	173 (37)	360 (53)	533
20–<50 K	199 (42)	234 (35)	433
50 K+	102 (22)	79 (12)	181
Missing (*n* = 11)			
Education			
≤High School	76 (16)	193 (28)	269
>High School	400 (84)	488 (72)	888
Missing (*n* = 1)			
Employed			
No	308 (65)	384 (56)	692
Yes	168 (35)	296 (44)	464
Missing (*n* = 2)			
Body Mass Index ^1^			
<25	127 (27)	108 (16)	235
25–29	90 (19)	157 (23)	247
30–34	92 (19)	124 (18)	216
35–40	76 (16)	113 (17)	189
40+	91 (19)	180 (26)	271
Heavy Alcohol Use (past year) ^2^			
No	403 (85)	530 (78)	933
Yes	73 (15)	152 (22)	225
Currently Married			
No	324 (68)	511 (75)	835
Yes	152 (32)	171 (25)	323
Current Use of Oral Contraception			
No	410 (86)	622 (91)	1032
Yes	66 (14)	60 (9)	126
Recent Birth			
<5 years	157 (33)	235 (34)	392
5+ years or no birth	319 (67)	447 (66)	766
Number of Births Prior to Last			
0–1 Birth	397 (83)	507 (74)	904
2 Births	57 (12)	87 (13)	144
3+ Births	22 (5)	88 (13)	110
Depo-Provera Use (Last 2 Years)			
No	426 (90)	615 (90)	1041
Yes	50 (11)	67 (10)	117
Age at Menarche (years)			
≤10	71 (15)	125 (18)	196
Age 11	99 (21)	139 (20)	238
Age 12	149 (31)	174 (26)	323
Age 13	73 (15)	115 (17)	188
14+	84 (18)	129 (19)	213
Current Smoker			
No	422 (89)	514 (75)	936
Yes	54 (11)	168 (25)	222
Number of Sex Partners			
0–5	194 (42)	145 (21)	339
6–10	132 (28)	200 (29)	332
≥11	139 (30)	335 (49)	474
Missing (*n* = 13)			
Age at 1st Intercourse (years)			
≤4	92 (20)	246 (36)	338
15–16	151 (32)	258 (38)	409
≥17 ^3^	222 (48)	175 (26)	397
Missing (*n* = 14)			
HSV-2 Seropositive			
No	318 (67)	288 (42)	606
Yes	157 (33)	392 (58)	549
Missing (*n* = 3)			

Abbreviations: HSV-2, herpes simplex virus type 2; ^1^ Body mass index was calculated using clinic-measure values as weight (kg)/height (m)^2^; ^2^ The alcohol-consumption variable reflected the drinking level each woman reported for the age(s) at which she was drinking the most. Heavy drinkers were those who usually consumed six or more drinks on days when they had alcohol or who consumed four or more drinks per sitting at least 2–3 times a month. ^3^ Includes participants who reported never having had sex.

**Table 2:** Genital *Chlamydia* serostatus and fibroid incidence among 1158 23- to 35-year-old black women with 2884 eligible follow-up visits across three follow-up intervals: hazard ratios and 95% confidence intervals.

Counts across 3 Follow-Up Intervals			Incident Fibroids		Unadjusted HR (95% CI)	Adjusted ^1^ HR (95% CI)
Chlamydia Serology	Women	Visits	*n*	% of Women		
Seronegative	476	1185	119	25	1.00 (ref)	1.00 (ref)
Seropositive	682	1699	158	23	0.9 (0.72, 1.16)	1.0 (0.79, 1.29)

Abbreviation: CI, confidence interval; HR, hazard ratio; ^1^ Adjusted for years since depo medroxyprogesterone acetate use, smoking, income, age at menarche, and recent birth and number of births prior to last birth (both anchored at the end of the interval); age is the time scale.

**Table 3:** The association between genital *Chlamydia* serostatus and fibroid growth per 18 months among fibroids from 395 black women with growth data for 1254 intervals of growth.

Chlamydia Serology	Growth Measurements ^1^ *n* = 1254	Adjusted ^2^ Estimated Percent Difference in Growth/18 Months (95% CI)
Seronegative	645	
Seropositive	609	4.4% (−5.02, 14.64) ^3^

Abbreviation: CI, confidence interval; ^1^ Growth is modelled as the difference in the natural log volume from 1 visit to the next visit, scaled to 18 months; ^2^ Adjusted for fibroid volume, fibroid number, age at the beginning of the interval, years since depo medroxyprogesterone acetate use, income, smoking, age at menarche, and number of births prior to last and recent birth anchored at the end of the interval; ^3^ An estimated percent growth difference of 4.4% indicates that the average growth (volume change per 18 months) for fibroids from *Chlamydia* seropositive women was an estimated 4.4% greater than that for fibroids from *Chlamydia* seronegative women.

## Data Availability

The data and computer code are not available for replication because the data are not publicly available. Data may be requested by contacting the Principal Investigator, Donna Baird, baird@niehs.nih.gov.
